# Supplementary material for: The Association between Plant-Based Diet Indices and Metabolic Syndrome in Chinese Adults: Longitudinal Analyses from the China Health and Nutrition Survey
Source: Nutrients. 2023 Mar 9;15(6):1341. doi: 10.3390/nu15061341 (PMC10057991; doi:10.3390/nu15061341)
Supplement: Supplementary file 1 [file nutrients-15-01341-s001.zip › nutrients-2264038-supplementary.pdf]

Supplementary data

Supplementary Table S1. The classification of food items in the China Health and Nutrition Survey (CHNS).

Plant Food Groups		***Food Codes (FD)***
Healthy		
Whole grains	Wheat, wheat germ flour, bran, black rice, brown rice, corn, barley, millet, yellow rice, cereal, oatmeal	011101 =<FD=< 011208 or<br>012001=<FD=<012306 or<br>013101 =<FD=<013201 or<br>014101 =<FD=<019014 or<br>FD=152102 or FD=152103
Fruits	kernel fruits, berry fruit, citrus, tropical and subtropical fruits, melon, fruit juice, melons	061101=<FD=<066206 or<br>162001 =<FD=<162028
Vegetables	Root vegetables, fresh beans, eggplant, garlic and onion, young stems and leaves, aquatic vegetables, potatoes and taro, wild vegetables, mushrooms and algae	041101=<FD=<048088 or<br>051001=<FD=<052011 or<br>021201=<FD=<021301
Nuts	Peanuts/almonds/pine nuts	071001=<FD=<072026
Legumes	Non- fermented bean products : tofu, soy milk powder, mung bean, red bean, broad bean, other beans	031101=<FD=<039902
Tea and coffee	Coffee, tea	166101=<FD=<166202 or<br>167016=<FD=<167018
Less healthy		
Refined grains	Noodles, white rice, bread, biscuits, and other leisure foods, flour, instant noodles, starch, rice cake/rice cake soup, other rice cakes	011301=<FD=<011503 or<br>012401=<FD=<012411 or

		FD=0132202 or FD=019201 or FD=152101 or 152104=<FD=<153002 or FD=153004 or 141001=<FD=<141037 or 022101=<FD=<022203
Potatoes	Potatoes, chips	021101=<FD=<021108 or FD=153003
Sugar sweetened beverages	Carbonated beverages, solid beverages, milk beverages, plant protein beverages, other beverages.	161001 =< FD=161008 or 163001=<FD=<165002 or 167001=<FD=<169013
Sweets and desserts	Cakes, desserts, mooncakes, sugars, preserves	142101=<FD=<142333 or 181001=<FD=<184005
Fermented food group	Bean sauce, peanut butter, salted vegetable, pickled vegetable (preserved in soy sauce or salt)	203101=<FD=<205048
Animal Food Groups		
Animal fat	Animal(pig, cattle and sheep) oils and fats	191001=<FD=<191007
Dairy	Milk, yogurt/yoplait, ice cream, cheese	101101=<FD=<109009
Eggs	Eggs/quail eggs	111101=<FD=<114201
Fish	Fish, shrimp, crab, shellfish and mollusc	121101=<FD=<129302
Meat	Subclasses of pig, sheep, donkey, horse, poultry, etc	081101=<FD=<089006 or 091101=<FD=<099004

Supplementary Table S2. Hazard ratios (95% CI) of plant-based diet indices and incident MetS after multiple imputation.

	Quintile 1	Quintile 2	Quintile 3	Quintile 4	Quintile 5	P-trend
		hPDI				
Crude model	Reference	0.91(0.87,0.94)	0.71(0.68,0.74)	0.64(0.61,0.68)	0.63(0.60,0.65)	<.0001
Adjusted model	Reference	0.94(0.91,0.98)	0.77(0.74,0.81)	0.72(0.68,0.76)	0.73(0.70,0.77)	<.0001
		uPDI				
Crude model	Reference	0.88(0.84,0.93)	0.95(0.91,0.98)	1.04(1.00,1.09)	1.08(1.03,1.12)	<.0001
Adjusted model	Reference	0.96(0.92,1.01)	1.05(1.01,1.10)	1.21(1.15,1.27)	1.26(1.21,1.32)	<.0001

Adjusted model was adjusted for age, sex, total energy intake(d/kcal), total carbohydrate intake(g), total fat intake(g), total protein intake(g), education, physical activity, smoking status, and alcohol intake. hPDI, health plant-based diet index; uPDI, unhealthful plant-based diet index.

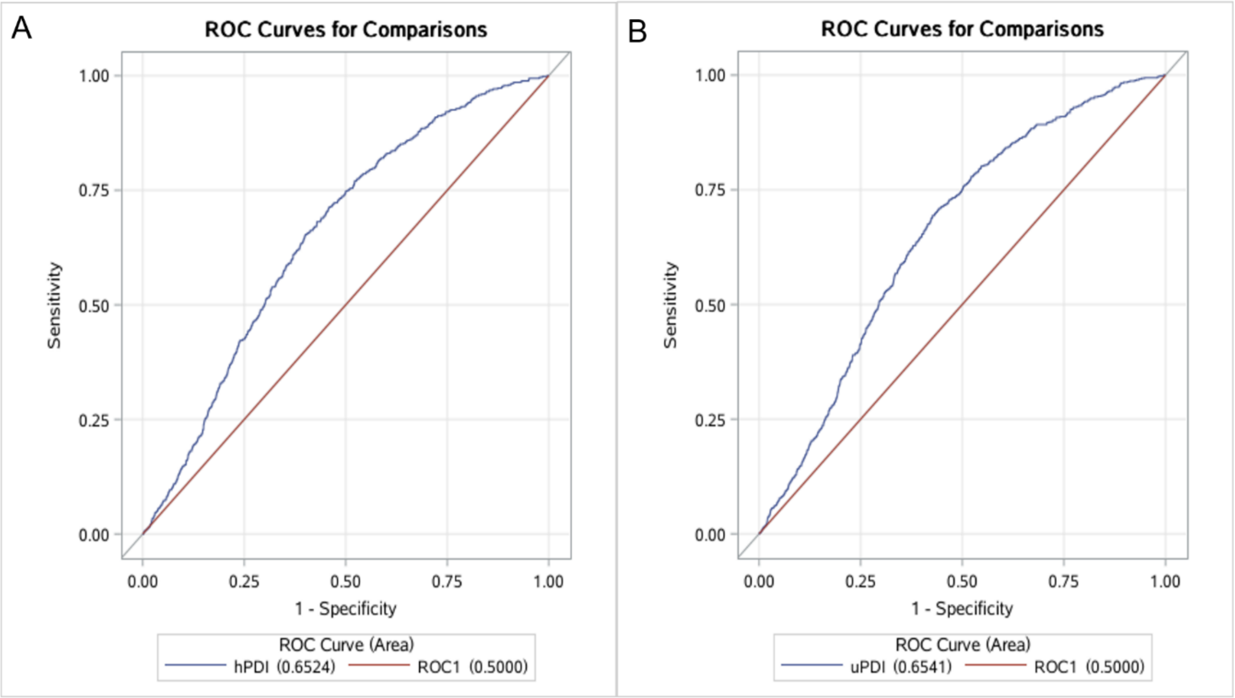

Supplementary Figure S1. The receiver operating characteristic curve (ROC) curves of hPDI (A) and uPDI (B). hPDI, healthful plant-based diet index; uPDI, unhealthful plant-based diet index.
